# Comparative genomic analysis of the *Pediococcus* genus reveals functional diversity for fermentation and probiotic applications

**DOI:** 10.1016/j.csbj.2025.10.050

**Published:** 2025-10-25

**Authors:** Nattarika Chaichana, Jirasa Boonsan, Thitaporn Dechathai, Sirikan Suwannasin, Kamonnut Singkhamanan, Monwadee Wonglapsuwan, Rattanaruji Pomwised, Komwit Surachat

**Affiliations:** a Department of Biomedical Sciences and Biomedical Engineering, Faculty of Medicine, Prince of Songkla University, Hat Yai , Songkhla 90110, Thailand; b Division of Biological Science, Faculty of Science, Prince of Songkla University, Hat Yai, Songkhla, Thailand

**Keywords:** *Pediococcus*, Comparative genomics, Pan-genome analysis, Probiotic genes, Biosynthesis gene clusters, Carbohydrate-active enzymes

## Abstract

*Pediococcus*, a genus of lactic acid bacteria (LABs), plays key roles in food fermentations yet remains underexplored at the genomic level. This study conducted a comparative genomic analysis of 616 *Pediococcus* genomes, providing the most comprehensive evaluation of this genus to date. Genome sizes ranged from 0.56 to 2.53 Mbp with distinct GC signatures, while ANI-based clustering and phylogenomic reconstruction resolved clear species boundaries and identified a potential novel lineage within the *P. acidilactici* complex, supported by 90 % ANI and 37–38 % digital DNA–DNA hybridization (dDDH) relative to the type strains. Pan-genome analysis identified 34,011 orthologous clusters but only 32 core genes, confirming an open pan-genome and remarkable genomic flexibility. In addition, safety assessment showed a low prevalence of antimicrobial resistance genes (6.66 %) and absence of classical virulence determinants, supporting the overall safety of the genus. Functional annotation revealed a diverse arsenal of bacteriocins and secondary metabolite clusters, with *P. pentosaceus* carrying the richest repertoire, including pediocin and penocin A. Carbohydrate-active enzymes (CAZymes), dominated by glycoside hydrolases (GH) and glycosyltransferases (GT), reflected adaptation to carbohydrate-rich niches. Importantly, probiotic-associated traits were broadly distributed but species-specific. *P. acidilactici* emerged as the most versatile, harboring genes for adhesion, acid-bile tolerance, oxidative stress defense, vitamin biosynthesis, and γ-aminobutyric acid (GABA) metabolism. These findings establish *Pediococcus* as a genus with low pathogenic risk, strong probiotic potential, and broad functional diversity. This genomic framework provides valuable guidance for the rational selection of strains in food fermentations, probiotic development, and biotechnological innovation.

## Graphical Abstract

**Figure:**
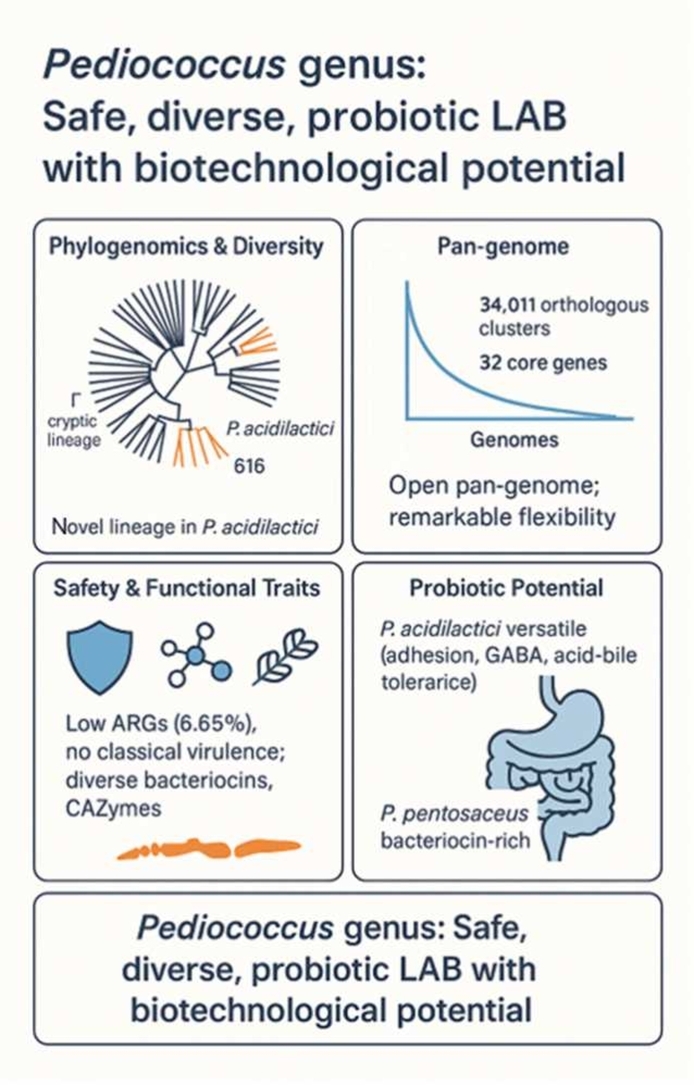

## 1 Introduction

Lactic acid bacteria (LAB) are a diverse group of Gram-positive microorganisms widely recognized for their roles in food fermentation, preservation, and human health. They are commonly found in fermented foods, plant materials, and animal microbiota, where they contribute to flavor development, nutritional enhancement, and safety through the production of antimicrobial compounds [105]. LAB are characterized by their ability to ferment carbohydrates anaerobically into lactate. They are classified into homofermentative species, which primarily produce lactate as the sole end product, and heterofermentative species, which additionally generate ethanol and carbon dioxide [1], [7]. The genus *Pediococcus*, within the family Lactobacillaceae, comprises several recognized species that exhibit diverse ecological niches and functional traits [64]. The most prevalent are *P. acidilactici* and *P. pentosaceus*, both widely associated with fermented foods and well-studied for their probiotic potential and production of pediocin-like bacteriocins [77]. Other species are more specialized, including *P. parvulus* and *P. ethanolidurans* produce β-glucan exopolysaccharides that influence alcoholic beverage texture [69], while *P. damnosus, P. inopinatus,* and *P. claussenii* are commonly linked to beer and wine aroma [12], [92]. Additional species, *P. cellicola* have been isolated from a distilled-spirit-fermenting cellar [118], and *P. argentinicus* is associated with Argentinean wheat flour [24]. Rare species, such as *P. siamensis* and *P. stilesii,* have been identified from Thai fermented foods and maize grains, respectively [32], [98]. These species highlight the dual role of *Pediococcus* as both beneficial microbes in food fermentation, probiotics, and other environmental niches. Most notably, *P. acidilactici* and *P. pentosaceus* are widely applied in food and feed industries due to their probiotic attributes, including tolerance to gastrointestinal stresses, adhesion capacity, production of vitamins, and antimicrobial activity through bacteriocin synthesis [13], [77], [97]. These bacteriocins, such as pediocin and pediocin-like, exhibit strong inhibitory activity against foodborne pathogens such as *Listeria monocytogenes*, highlighting their value as natural biopreservatives [33], [44]. In addition to food applications, *Pediococcus* has gained attention for potential therapeutic uses, including modulation of the gut microbiota and enhancement of host immunity [28], [74].

Despite their industrial and health relevance, genome-wide comparative analyses of *Pediococcus* remain limited. Most available studies have focused on single strains or small species groups, leaving the genus-level diversity, evolutionary structure, and functional potential largely unresolved. To address this gap, we conducted a comprehensive comparative genomic analysis of 616 *Pediococcus* genomes. The study aims to (i) delineate species boundaries using average nucleotide identity (ANI) and phylogenomics, (ii) explore the pan-genome structure and genomic diversity, and (iii) investigate functional traits related to safety, carbohydrate metabolism, secondary metabolite biosynthesis, and probiotic potential. By integrating large-scale comparative and functional analyses, this work provides the most extensive genomic framework to date for understanding the ecological and biotechnological significance of the *Pediococcus* genus.

## 2 Materials and methods

### 2.1 Data collection and genome annotations

All 616 *Pediococcus* genomes available in the NCBI database were retrieved (accessed on 01 August 2025). Genome annotation was performed using the rapid prokaryotic genome annotation (Prokka 1.14.6) [87].

### 2.2 Species identification and pairwise average nucleotide identity (ANI) values among *Pediococcus* strains

All *Pediococcus* genomes were re-annotated for taxonomy, and the species were identified in this genus. To assess the ANI values across all *Pediococcus* species genomes, a total of 616 genomes were subjected to pairwise comparison using FastANI v1.34 [42]. The potential novel species with a cryptic lineage were analyzed digital DNA-DNA hybridization (dDDH) values using the Genome-to-Genome Distance Calculator (GGDC).

### 2.3 Detection of antimicrobial resistance genes and virulence-associated genes

The presence of antimicrobial resistance genes (ARGs) in *Pediococcus* species genomes was detected using ABRicate (v1.0.1) with default parameters against the comprehensive antibiotic resistance database (CARD) [2]. In addition, potential virulence-associated genes were searched using the virulence factor database (VFDB) [54] with an identity > 80 % to compare against the reference virulence factors of pathogenic bacteria.

### 2.4 Pan-genome and comparative analysis

The pan-genome analysis of all 616 *Pediococcus* strains was constructed using the Roary pipeline [71], where proteins with ≥ 95 % amino acid sequence identity were grouped into the same orthologous family. A maximum-likelihood phylogenomic tree was generated using FastTree v2.1 [78] and subsequently annotated and visualized using the Interactive Tree of Life (iTOL) v8.

### 2.5 Identification of bacteriocin and secondary metabolite biosynthesis gene (BGC) clusters

To identify BGCs associated with bacteriocins and secondary metabolites in *Pediococcus* genomes, BAGEL4 was employed using Hidden Markov Model (HMM)-based searches against curated bacteriocin databases, enabling precise detection and functional classification of putative bacteriocin gene clusters [102]. The antiSMASH v8.0 was executed with default parameters, which include automated detection of all major BGC types (RiPPs, NRPS, PKS, terpenes, and hybrids) and comparison against the MIBiG database [15].

### 2.6 Prediction of Carbohydrate-Active Enzyme (CAZY) family

The active genes for the enzymes of carbohydrates in the *Pediococcus* species genomes were identified using dbCAN3 with the DIAMOND tool against the CAZy database [123]. The database primarily categorizes enzymes into six major groups, including glycoside hydrolases (GHs), glycosyltransferases (GTs), carbohydrate esterases (CEs), carbohydrate-binding modules (CBMs), auxiliary activity enzymes (AAs), and polysaccharide lyases (PLs).

### 2.7 Probiotic gene identification and enrichment analysis

Probiotic-associated genes, including gastrointestinal tract (GIT) tolerance, adhesion ability, BGC metabolite synthesis, the GABA system, stress resistance, vitamin synthesis, and other mechanisms of oxidative stress resistance, were identified across isolates and mapped to species metadata to calculate prevalence using the gene presence and absence information from Roary.

To assess species-specific enrichment of probiotic-related traits, we performed Fisher’s exact tests for each functional gene category across *Pediococcus* species. Presence/absence of each gene category in the species was compared against all other species in the dataset to determine whether the gene category’s distribution was non-randomly associated with any specific species. The odds ratio (OR) was calculated to quantify the strength and direction of association, with values greater than 1 indicating enrichment and values less than 1 indicating depletion in the species. Moreover, p-values were corrected for multiple comparisons using the Benjamini–Hochberg false discovery rate (FDR) method, and q-values less than 0.05 were considered statistically significant. A functional clustering score was computed by summing the -log_10_(q-value) across all significant gene categories (q < 0.05), reflecting the overall functional richness in each species.

### 2.8 Data visualization and statistical analysis

All information on species-level strengths was then summarized, and visualization was performed using Python v3.13.7.

## 3 Results

### 3.1 Genomic information of *Pediococcus* isolates

**Fig. 1:**
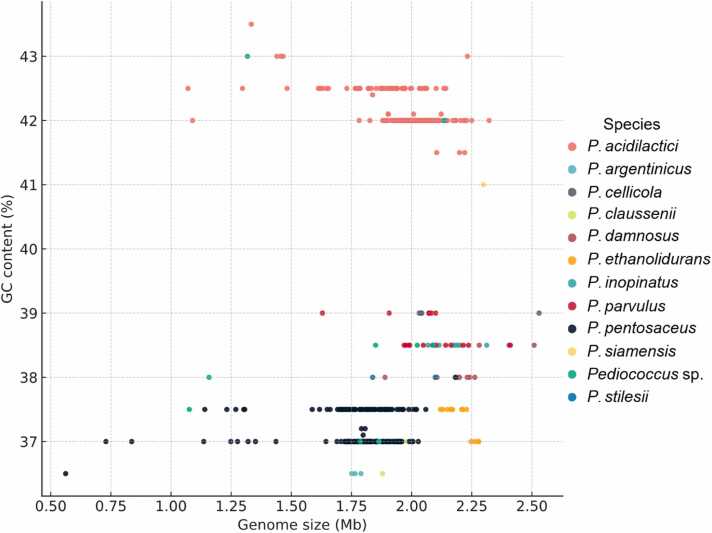
Genome size-GC content landscape of 616 *Pediococcus* genomes. Colors represent species and shapes indicate assembly level. The two dominant clusters correspond to *P. acidilactici* and *P. pentosaceus*, while smaller groups denote other species.

In this study, 616 *Pediococcus* strains were retrieved from the NCBI database, and their genome information was summarized to provide an overview of the dataset. The genome sizes ranged from 0.56 to 2.53 Mbp, with most genomes distributed between 1.8 and 2.1 Mbp (Figure S1A). The GC content varied from 36.5 % to 43.5 %, showing two prominent peaks at approximately 37 % and 42 % (Figure S1B). The number of contigs ranged widely from 1 to 1897, although the majority of genomes contained fewer than 50 contigs (Figure S1C). With respect to assembly level, more than half of the genomes were at the contig level (350 genomes, 56.8 %), followed by scaffold assemblies (137 genomes, 22.2 %), complete assemblies (123 genomes, 20.0 %), and a small number classified at the chromosome level (6 genomes, 1.0 %) (Figure S1D). A total of 616 *Pediococcus* genomes were retrieved and classified at the species level. Among these, *P. acidilactici* was the most represented species with 335 genomes, followed by *P. pentosaceus* with 204 genomes. Other species were less frequently observed, including *P. parvulus* (18 genomes), *P. ethanolidurans* (13 genomes), and *P. damnosus* (13 genomes). In addition, 10 genomes were identified as unclassified *Pediococcus* sp., while smaller numbers were detected for *P. inopinatus* (8 genomes), *P. argentinicus* (4 genomes), *P. claussenii* (4 genomes), and *P. cellicola* (4 genomes). Rare species such as *P. stilesii* (2 genomes) and *P. siamensis* (1 genome) were also present in the dataset. Distinct patterns of genome features were observed among the 616 *Pediococcus* strains (Fig. 1). The two dominant clusters, corresponding to *P. acidilactici* and *P. pentosaceus*, displayed compact and well-defined ranges of genome size and GC content. In contrast, smaller species such as P*. parvulus, P. damnosus,* and *P. ethanolidurans* exhibited narrower but distinct compositional signatures. Notably, a subgroup of 13 *P. acidilactici* strains showed slightly elevated GC content (approximately 1.5 % higher) and smaller genome sizes compared with the main *P. acidilactici* cluster, forming a separate compositional group.

### 3.2 Pairwise ANI values of *Pediococcus* strains

**Fig. 2:**
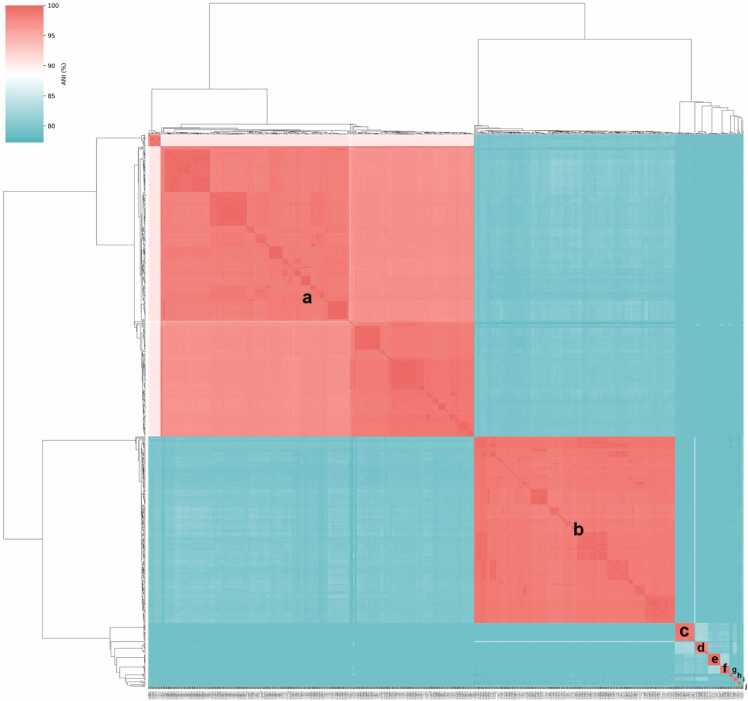
Heatmap of pairwise ANI among 616 *Pediococcus* genomes. ANI values range from below 80–100 %, visualized with a gradient scale from cyan (low identity) to red (high identity). Hierarchical clustering based on ANI clearly resolves two major clades, labeled a and b, corresponding to *P. acidilactici* and *P. pentosaceus*, respectively. Additional minor clusters (c-j) contain species such as *P. parvulus* (clade c), *P. ethanolidurans* (clade d), *P. damnosus* (clade e), *P. inopinatus* (clade f), *P. claussenii* (clade g), *P. cellicola* (clade h), *P. argentinicus* (clade i), and *P. stilesii* (clade j).

Pairwise ANI analysis among the 616 *Pediococcus* genomes revealed values ranging from below 80–100 % (Fig. 2), reflecting variation across species within the genus. ANI-based clustering resolved two major clades corresponding to *P. acidilactici* (clade a) and *P. pentosaceus* (clade b). Within the *P. acidilactici* clade, a distinct subgroup of 13 strains showed approximately 90 % ANI relative to typical *P. acidilactici* genomes, below the 95 % threshold for species-level cutoff. The *P. pentosaceus* genomes formed a coherent cluster with ANI values above 95 %. A smaller group, including *P. parvulus* (clade c), *P. ethanolidurans* (clade d), *P. damnosus* (clade e), *P. inopinatus* (clade f), *P. claussenii* (clade g), *P. cellicola* (clade h), *P. argentinicus* (clade i), and *P. stilesii* (clade j), formed a separate cluster. Within this cluster, intra-species ANI values exceeded 95 %, while inter-species comparisons ranged from 85 % to 92 %. ANI values among this cluster and the major clades were generally below 85 %, confirming species-level separation and distinct lineages. Consistent with the ANI results, dDDH analysis further supported the distinctiveness of this subgroup within *P. acidilactici*. The 13 divergent genomes exhibited dDDH values ranging from 37.0 % to 38.4 % when compared with the closest *P. acidilactici* strains, which are well below the 70 % species delineation threshold.

### 3.3 Safety evaluation

**Fig. 3:**
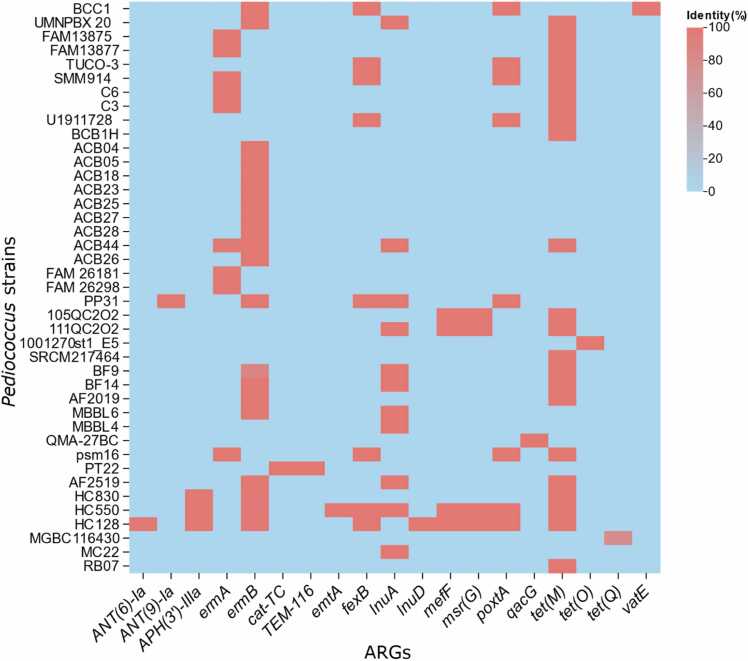
Heatmap showing the distribution of ARGs across *Pediococcus* genomes. Each row represents an individual strain, and each column corresponds to a specific ARG, represented by the identity (%) of each gene.

The safety evaluation was carried out by screening all 616 *Pediococcus* genomes for the presence of ARG and virulence factor-associated (VF) genes. Analysis of ARGs revealed that only 41 out of 616 strains (6.66 %) carried ARGs in their genomes. Several ARGs were detected across multiple strains, indicating a broader distribution within the genus, whereas others appeared only in one or a few isolates. The number of ARGs varied by strain, ranging from none to multiple genes. The strains HC550 and HC128, both isolated from animals, harbored the highest number of ARGs, with nine each. Among all identified ARGs, *tet(M)* was the most prevalent, followed by *ermB* and *lnuA* (Fig. 3).

**Fig. 4:**
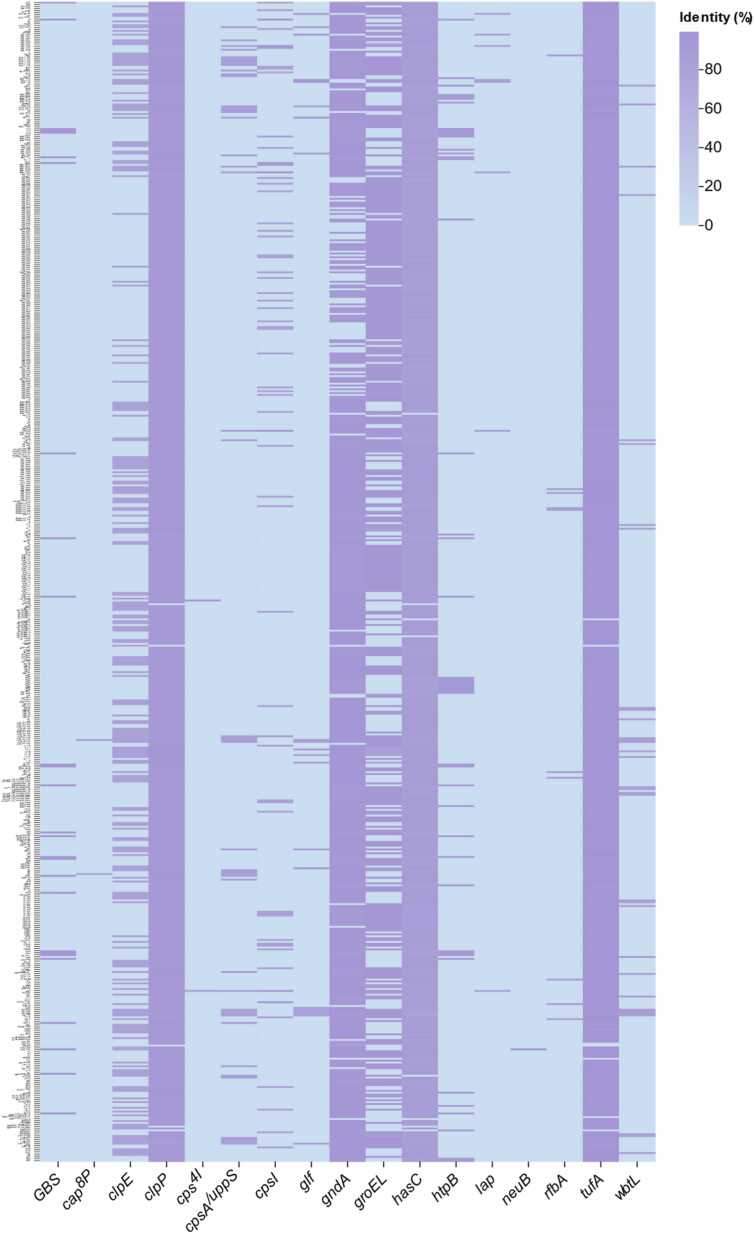
Heatmap of VF genes in 616 *Pediococcus* genomes. Rows represent genomes and columns represent VF genes, with color intensity indicating sequence identity (%).

Virulence gene profiling across 615 of the 616 *Pediococcus* genomes (99.84 %) revealed that nearly all strains carried at least one virulence factor (VF) gene. The number of VF genes varied among strains, ranging from only a few to multiple determinants. Strain CIRM-BIA 750 contained the highest number, with 10 VF genes, followed by strains CIRM-BIA 2166, FUA 30086, FUA 30084, FUA 30085, and FUA 3699, each carrying nine genes. The remaining strains are typically harbored between five and eight VF genes. The most frequently detected VF genes across the genus were *clpP*, *gndA*, *hasC*, and *tufa*, while other genes were distributed in a strain-specific manner (Fig. 4).

### 3.4 Pan-genome and comparative analysis

**Fig. 5:**
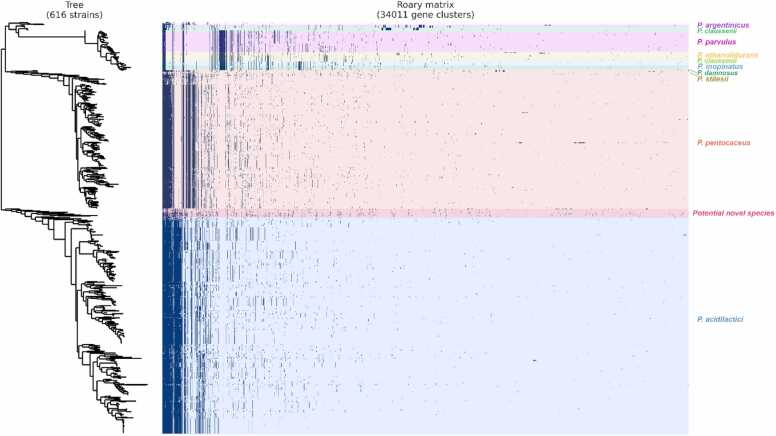
Pan-genome analysis of 616 *Pediococcus* strains showing the Roary gene presence or absence matrix and phylogenomic tree based on SNPs in core genes. The matrix displays 34,011 gene clusters, with species-specific clustering observed.

Pan-genome analysis identified 34,011 gene clusters across 616 genomes. Core genes, present in 99–100 % of strains, numbered only 32 genes. Soft-core genes (127 genes) were present in 95–99 % of genomes, shell genes (2783 genes) in 15–95 % of genomes, and cloud genes (31,069 genes) in less than 15 % of genomes (Figure S2A). Furthermore, the pan-genome frequency plot for the *Pediococcus* genus highlights an open pan-genome with considerable genomic diversity. The plot shows that a large number of gene families are present in only a few genomes, particularly in the leftmost region, indicating a high proportion of strain-specific cloud genes. The number of genes shared across multiple genomes decreases significantly, indicating that the core genome is relatively small and consists of a few universally conserved genes (Figure S2B). Pan-genome accumulation curve increases steadily as more genomes are added, whereas the core genome curve rapidly plateaus, showing that only a small set of genes is shared among all genomes (Figure S2C). The pan-genome phylogenomic tree visualized through a Roary gene presence/absence matrix and a phylogenomic tree based on SNPs in accessory core genes, reveals clear species-level clustering. The result reveals distinct clades that separate *Pediococcus* species, with two large groups corresponding to *P. acidilactici* and *P. pentosaceus*, each showing dense vertical blocks of shared core genes. In contrast, smaller species such as *P. argentinicus*, *P. claussenii*, *P. parvulus*, *P. ethanolidurans*, *P. inopinatus*, *P. damnosus*, and *P. stilesii* form smaller clusters, as shown in the phylogenomic tree constructed from SNP core gene information. Interestingly, a distinct clade labeled as a potential novel species is observed between the well-established *P. pentosaceus* and *P. acidilactici* clades. The accompanying heatmap illustrates the presence and absence of gene clusters across the genomes, encompassing a total of 34,011 genes (Fig. 5).

### 3.5 Phylogenomic comparison of *Pediococcus* strains

**Fig. 6:**
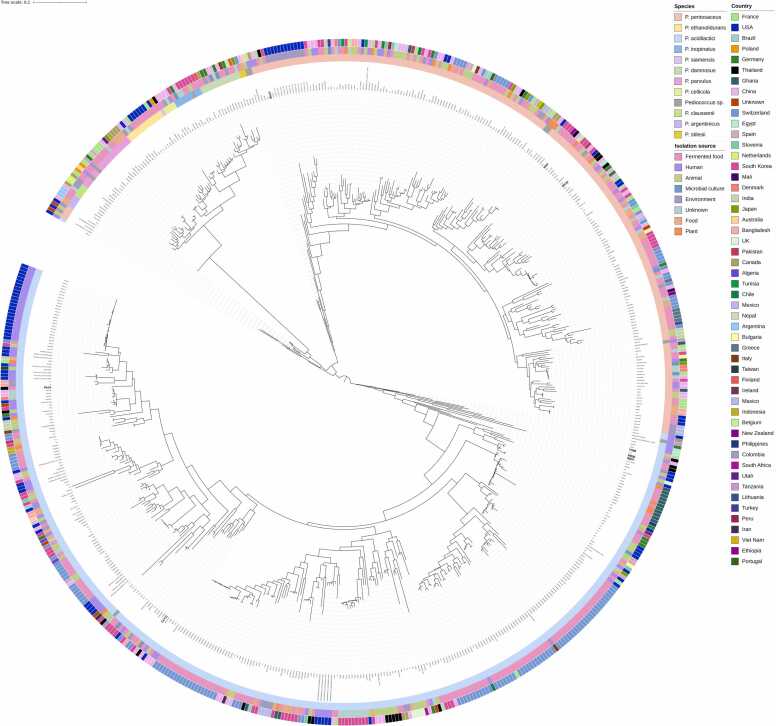
Phylogenomic tree of 616 *Pediococcus* genomes based on core gene SNPs. The tree resolves clear species-level clusters within the genus. Two dominant clades correspond to *P. acidilactici* and *P. pentosaceus*, while smaller, well-defined clusters represent other species, including *P. parvulus*, *P. ethanolidurans, P. damnosus, P. inopinatus, P. argentinicus, P. claussenii, P. cellicola, P. stilesii*, and *P. siamensis*. A distinct branch between *P. acidilactici* and *P. pentosaceus* suggests the presence of a potential novel lineage. The outer concentric rings indicate the innermost species classification, with each color representing a different *Pediococcus* species. This is followed by the isolation source, categorized as food, fermented food, animal, human, environment, or microbial culture. The outermost ring indicates the country of origin for each genome, illustrating the broad global distribution of *Pediococcus* isolates.

The phylogenomic analysis of 616 *Pediococcus* genomes clearly resolved species-level classification, with the outermost ring of the tree highlighting distinct clusters corresponding to recognized species. The two largest groups were *P. acidilactici* (335 genomes) and *P. pentosaceus* (204 genomes), which formed well-defined clades representing the dominant taxa within the genus. Smaller clusters were observed for *P. parvulus* (18 genomes), *P. ethanolidurans* (13 genomes), *P. damnosus* (13 genomes), *P. inopinatus* (8 genomes), *P. argentinicus* (4 genomes), *P. claussenii* (4 genomes), *P. cellicola* (4 genomes), *P. stilesii* (2 genomes), and *P. siamensis* (1 genome). A further 10 genomes were classified as unassigned *Pediococcus sp.*, with several branching between the two major clades. Food-associated isolates dominated the dataset, particularly in *P. acidilactici* and *P. pentosaceus*, which are widely linked to fermented food products. Strains associated with beer and wine fermentations were prominent among *P. parvulus, P. damnosus,* and *P. ethanolidurans*, reflecting their adaptation to alcoholic beverage environments. Human- and animal-derived isolates were mostly observed within *P. acidilactici* and *P. pentosaceus*, while a smaller number of strains originated from plants, the environment, or microbial culture collections. Geographic distribution revealed the cosmopolitan nature of *P. acidilactici* and *P. pentosaceus*, which were recovered from multiple countries across Asia, Europe, North America, and South America, including the USA, France, Brazil, Germany, Thailand, China, and Japan. In contrast, minor species such as *P. parvulus, P. damnosus,* and *P. ethanolidurans* displayed narrower distributions, being largely confined to European countries with a history of beer and wine production. Rare taxa such as *P. siamensis* and *P. cellicola* were limited to single-country origins (Fig. 6).

### 3.6 BGCs for secondary metabolites and bacteriocins

**Fig. 7:**
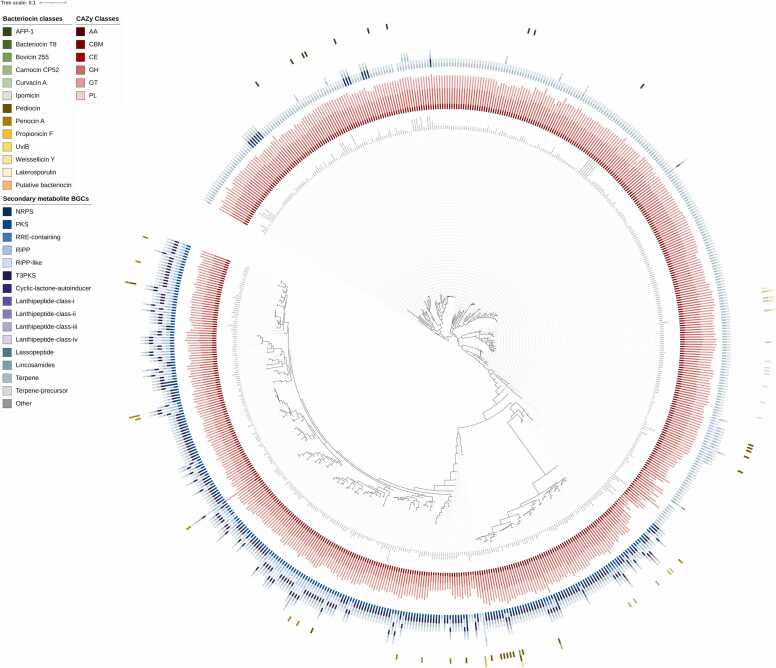
Distribution of bacteriocins, secondary metabolite BGCs, and CAZyme families across 616 *Pediococcus* genomes. Bacteriocin profiles show that pediocin, and penocin A, and AFP-1 are the most prevalent. Secondary metabolite BGCs identified by antiSMASH reveal conserved terpene clusters across all species, while PKS/T3PKS and RiPP-like clusters display species-specific enrichment.

**Fig. 8:**
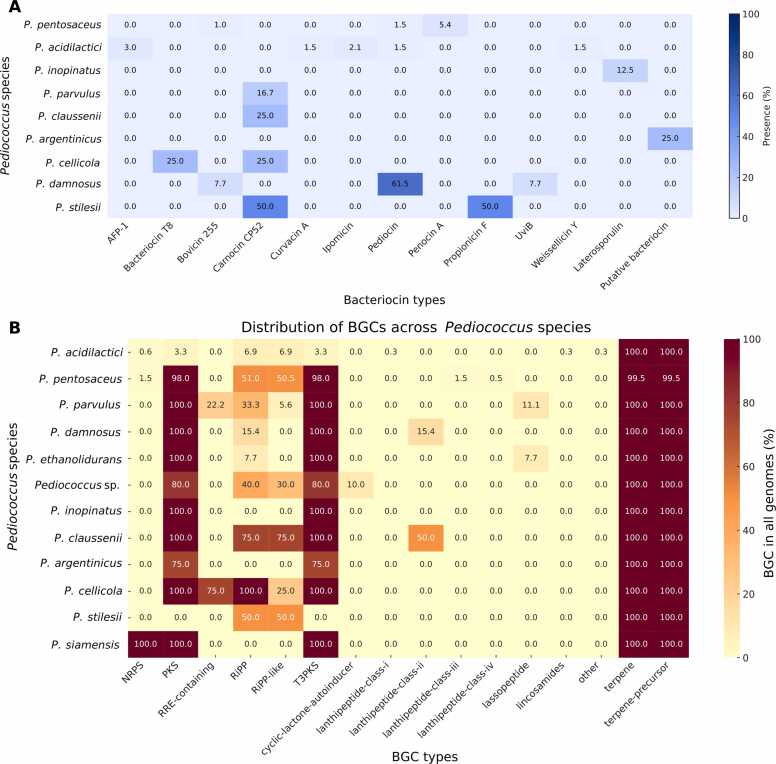
Heatmap distribution of bacteriocin and secondary metabolite BGCs across *Pediococcus* species. The prevalence of complete bacteriocin types across species (A). The distribution of secondary metabolite BGCs (B).

The distribution of bacteriocins and secondary metabolite BGCs across the *Pediococcus* phylogeny revealed clear clade-specific patterns. The species-level distribution of complete bacteriocin BGCs revealed that only a subset of species contained these clusters. *P. pentosaceus* and *P. acidilactici* displayed the greatest diversity, although their overall presence was low. Pediocin complete clusters were detected in approximately 1.5 % of *P. pentosaceus* and 1.5 % of *P. acidilactici* genomes, while bovicin 255 occurred in 1.0 % and propionicin F in 5.4 % of *P. pentosaceus* genomes. *P. acidilactici* also harbored anti-*Listeria* factor from *Pediococcus* or AFP-1 (3.0 %), curvacin A (1.5 %), and pediocin (1.5 %) clusters. *P. damnosus* exhibited the highest proportion of complete pediocin clusters (61.5 %), together with bovicin 255 (7.7 %) and UV-inducible bacteriocin or UviB (7.7 %). In addition, *P. stilesii* carried carnocin CP52 and propionicin F clusters in 50 % of genomes each. *P. cellicola* showed bovicin 255 and carnocin CP52 clusters in 25 % of genomes, while *P. claussenii* harbored carnocin CP52 in 25 %. *P. argentinicus* contained a putative bacteriocin cluster in 25 % of genomes. Other species, including *P. parvulus* and *P. inopinatus*, showed limited detection of complete bacteriocin clusters, with most clusters absent or below 25 %. Species with no complete bacteriocin clusters were excluded from the figure. However, *P. siamensis, P. ethanolidurans*, and unclassified *Pediococcus* sp. were absent complete bacteriocin BGCs in any genome (Figs. 7 and 8A).

In addition, the distribution of secondary metabolite BGCs identified by antiSMASH across *Pediococcus* species revealed both conserved and species-specific patterns (Fig. 7). Terpene and terpene-precursor clusters were universally conserved, detected in nearly all strains across every species, highlighting their role as core features of the genus. In contrast, polyketide synthase (PKS) and type III polyketide synthase (T3PKS) clusters showed strong lineage bias, being highly enriched in *P. pentosaceus* and consistently present in minor species such as *P. parvulus*, *P. damnosus*, *P. ethanolidurans*, *P. inopinatus*, and *P. claussenii*, but rare in *P. acidilactici*. Ribosomally synthesized and post-translationally modified peptides (RiPP) and RiPP-like clusters occurred at moderate frequencies, including 51.0 % and 50.5 % of *P. pentosaceus*, 75.0 % of *P. claussenii*, and 50.0 % of *P. stilesii*. By contrast, non-ribosomal peptide synthetase (NRPS), RiPP recognition element (RRE-containing), and lanthipeptide subclasses (I-IV) were only sporadically detected, generally restricted to individual strains or small subclades. The heatmap distribution of these BGCs across *Pediococcus* species is illustrated in Fig. 8B.

### 3.7 Distribution of CAZyme families among *Pediococcus* species

**Fig. 9:**
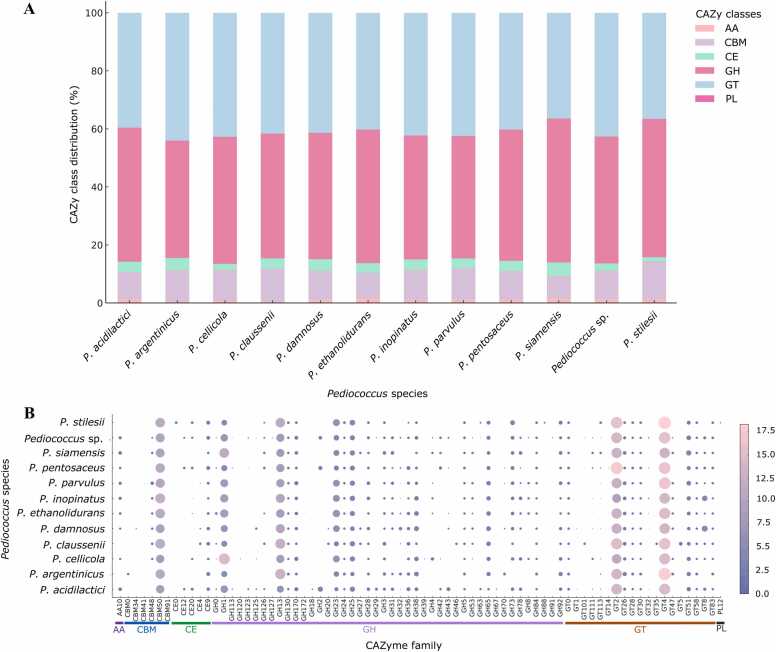
CAZyme class composition and family distribution across *Pediococcus* species.

CAZyme composition across *Pediococcus* species highlighted key enzyme families. The analysis revealed distinct patterns across six major enzyme classes, including GH, GT, CBM, CE, AA, and PL. GH and GT families accounted for the largest proportion of CAZymes in nearly all species analyzed. Specifically, *P. acidilactici*, *P. pentosaceus*, and *P. damnosus,* primarily isolated from traditional fermented food sources, exhibited a greater abundance of GH families, comprising between 45 % and 61 % of their total CAZyme content. GT families were consistently present across all species, representing between 30 % and 45 % of the total CAZy profile. CBM families showed moderate representation (8–12 %), with no strong species-level variation. In contrast, CE and AA families were found at lower frequencies, often below 5 %, and were mainly detected in a limited number of species such as *P. argentinicus* and *P. claussenii*. PL families were absent or nearly undetectable in most genomes. Notably, strains derived from fermented food environments exhibited richer and more functionally diverse CAZyme repertoires than those isolated from animal feces (Fig. 9A). In addition, the CAZyme analysis revealed that GH and GT were the dominant enzyme classes across the *Pediococcus* genus (Fig. 9B). Among GH families, GH1, GH13, and GH23 were frequently detected across multiple species. Many other GH families, such as GH24, GH25, GH38, GH43, and GH70, were rarely detected and appeared to be species-specific. The GT2, GT4, and GT8 families were consistently present in nearly all species. Species such as *P. acidilactici* and *P. pentosaceus* exhibited the broadest CAZyme repertoires, whereas others, including *P. stilesii*, showed a more restricted enzyme profile. AA10, CE0, and CE4 occurred sporadically, and PL was rarely detected family.

Stacked bar plot showing the percentage of six CAZyme classes identified in each species (A). Bubble plot indicating the abundance of individual CAZyme families, with bubble size and color representing copy number (B).

### 3.8 Probiotic-associated genes among *Pediococcus* species

Comparative genomic analysis revealed a diverse repertoire of probiotic-related genes distributed across *Pediococcus* species. These genes were classified into key functional categories, including adhesion, GABA system, GIT tolerance, oxidative stress response, other stress tolerance, and vitamin biosynthesis (Table 1). Among these, adhesion-related genes, such as *cna* (collagen adhesin) and *sdrE* (surface protein), were identified across multiple species, supporting the potential for host interaction and colonization. The GABA system was represented by *gadB* (glutamate decarboxylase), *gadC* (glutamate/GABA antiporter), and *puuD* (γ-glutamyl-γ-aminobutyrate hydrolase PuuD), which were also present in several strains, indicating the genetic basis for GABA metabolism. Genes associated with GIT tolerance included the arginine deiminase (ADI) pathway (*arcA, arcB, arcD),* ATP synthase subunits (*atpA, atpB, atpC, atpD, atpE, atpF, atpG, atpH*), acid resistance-related genes (*dltA, dltC, dltD*), and bile salt tolerance genes (*bshA*), found broadly across species, except that *bshB1* was distributed only in the *P. damnosus.* A wide array of oxidative stress-related genes was detected, which were present in multiple species, supporting defense against reactive oxygen stress, while other stress tolerance genes highlighted the presence of stress-protective mechanisms such as heat/cold shock defense across *Pediococcus* isolates. Moreover, numerous genes involved in vitamin biosynthesis pathways were identified, including riboflavin (*ribA-E*), folate (*folP, folC, folE*), and thiamine (*thiD, thiE, thiF, thiG, thiM*) biosynthesis genes, which were detected across species and indicate the genetic potential for vitamin production among this genus.

**Table 1:** Probiotic-associated genes identified in *Pediococcus* genomes.

Gene category	Gene name	Function	*Pediococcus* species	Reference
Adhesion	*cna*	Collagen adhesin	*P. acidilactici, P. pentosaceus, P. damnosus*	Madani et al., [62]
	*srtA*	Sortase A	*P. cellicola, P. damnosus, P. parvulus, P. ethanolidurans, P. inopinatus, Pediococcus* sp., *P. stilesii, P. argentinicus*	Wu et al., [109]
GABA system	*gadB*	Glutamate decarboxylase	*P. acidilactici*	Lyu et al., [60]
	*gadC*	Putative glutamate/gamma-aminobutyrate antiporter	*P. acidilactici, P. claussenii, P. pentosaceus, P. siamensis, P. ethanolidurans, P. damnosus, P. argentinicus, Pediococcus* sp.	Banerjee et al., [11]
	*puuD*	Gamma-glutamyl-gamma-aminobutyrate hydrolase PuuD	*P. claussenii, P. parvulus*	Liu et al., [56]
GIT tolerance	*arcA*	Arginine deiminase	*P. acidilactici, P. pentosaceus, Pediococcus* sp.*., P. stilesii, P. siamensis*	Yang et al., [111]
	*arcB*	Ornithine carbamoyltransferase, catabolic	*P. pentosaceus, P. stilesii, Pediococcus* sp.	Ariute et al., [8]
	*arcD*	Arginine/ornithine antiporter	*P. siamensis*	[101]
	*atpA*	ATP synthase subunit alpha	*P. acidilactici, P. argentinicus, P. cellicola, P. claussenii, P. damnosus, P. ethanolidurans, P. inopinatus, P. parvulus, P. pentosaceus, P. siamensis, Pediococcus* sp.*, P. stilesii*	Wu et al., [107]
	*atpB*	ATP synthase subunit a	*P. acidilactici, P. argentinicus, P. cellicola, P. claussenii, P. damnosus, P. ethanolidurans, P. inopinatus, P. parvulus, P. siamensis, Pediococcus* sp.	Chen et al., [21]
	*atpC*	ATP synthase epsilon chain	*P. acidilactici, P. argentinicus, P. cellicola, P. claussenii, P. damnosus, P. ethanolidurans, P. inopinatus, P. parvulus, P. pentosaceus, P. siamensis, Pediococcus* sp.*, P. stilesii*	Cotter, Hill [22]
	*atpD*	ATP synthase subunit beta	*P. acidilactici, P. pentosaceus, P. stilesii, P. argentinicus, P. cellicola, P. claussenii, P. damnosus, P. ethanolidurans, P. inopinatus, P. parvulus, P. siamensis, Pediococcus* sp.	Duary et al., [30]
	*atpE*	ATP synthase subunit c	*P. acidilactici, P. argentinicus, P. cellicola, P. claussenii, P. damnosus, P. ethanolidurans, P. inopinatus, P. parvulus, P. pentosaceus, P. siamensis, Pediococcus* sp.*, P. stilesii*	Duary et al., [30]
	*atpF*	ATP synthase subunit b	*P. acidilactici, P. argentinicus, P. claussenii, P. pentosaceus, Pediococcus* sp.*, P. stilesii, P. cellicola, P. damnosus, P. ethanolidurans, P. inopinatus, P. parvulus, P. siamensis*	Duary et al., [30]
	*atpG*	ATP synthase gamma chain	*P. acidilactici, P. argentinicus, P. cellicola, P. damnosus, P. ethanolidurans, P. inopinatus, P. parvulus, P. pentosaceus, P. siamensis, Pediococcus* sp.	Duary et al., [30]
	*atpH*	ATP synthase subunit delta	*P. acidilactici, P. argentinicus, P. pentosaceus, Pediococcus* sp.*, P. stilesii, P. cellicola, P. damnosus, P. ethanolidurans, P. inopinatus, P. parvulus, P. siamensis, P. claussenii*	Duary et al., [30]
	*bshA*	N-acetyl-alpha-D-glucosaminyl L-malate synthase	*P. acidilactici, P. inopinatus, P. parvulus*	Tang et al., [100]
	*bshB1*	N-acetyl-alpha-D-glucosaminyl L-malate deacetylase 1	*P. damnosus*	Tang et al., [100]
	*dltA*	D-alanine--D-alanyl carrier protein ligase	*P. acidilactici, P. pentosaceus, Pediococcus* sp.*, P. stilesii, P. damnosus, P. inopinatus, P. cellicola, P. ethanolidurans, P. parvulus, P. siamensis*	Nikolopoulos et al., [68]
	*dltC*	D-alanyl carrier protein	*P. acidilactici, P. argentinicus, P. cellicola, P. claussenii, P. damnosus, P. ethanolidurans, P. inopinatus, P. parvulus, P. pentosaceus, P. siamensis, Pediococcus* sp.*, P. stilesii*	Nikolopoulos et al., [68]
	*dltD*	Protein DltD	*P. acidilactici, P. pentosaceus, Pediococcus* sp.*, P. cellicola, P. damnosus, P. ethanolidurans, P. inopinatus, P. parvulus, P. siamensis, P. claussenii*	Nikolopoulos et al., [68]
Oxidative stress	*bsaA*	Glutathione peroxidase BsaA	*P. cellicola, P. damnosus, P. ethanolidurans, P. inopinatus, P. parvulus, P. siamensis, Pediococcus* sp.	Podkowik et al., [76]
	*cydA*	Cytochrome bd ubiquinol oxidase subunit 1	*P. claussenii*	Borisov et al., [16]
	*cydB*	Cytochrome bd-I ubiquinol oxidase subunit 2	*P. acidilactici, P. claussenii*	Borisov et al., [16]
	*ftrV*	Ferredoxin-thioredoxin reductase, variable chain	*P. acidilactici*	Balsera et al., [10]
	*glpO*	Alpha-glycerophosphate oxidase	*P. acidilactici, P. pentosaceus, Pediococcus* sp.*, P. argentinicus, P. stilesii*	Andreevskaya et al., [6]
	*hmo*	4-hydroxymandelate oxidase	*P. acidilactici, P. pentosaceus, Pediococcus* sp.*, P. stilesii, P. argentinicus,*	Liu et al., [55]
	*katA*	Catalase	*P. acidilactici, P. sp.*	Bryukhanov et al., [19]
	*mco*	Multicopper oxidase mco	*P. acidilactici, P. pentosaceus, Pediococcus* sp.*, P. cellicola, P. damnosus, P. ethanolidurans, P. inopinatus, P. parvulus, P. siamensis, P. claussenii, P. argentinicus*	Pei et al., [73]
	*msrA*	Peptide methionine sulfoxide reductase MsrA	*P. acidilactici, P. argentinicus, P. claussenii, P. inopinatus, P. pentosaceus, Pediococcus* sp.*, P. stilesii, P. damnosus, P. parvulus, P. cellicola, P. ethanolidurans, P. siamensis*	(Lourenço [58])
	*msrB*	Peptide methionine sulfoxide reductase MsrB	*P. acidilactici, P. cellicola, P. damnosus, P. ethanolidurans, P. parvulus, P. pentosaceus, P. siamensis, Pediococcus* sp., *P. argentinicus, P. claussenii, P. inopinatus, P. stilesii*	(Lourenço [58])

	*namA*	NADPH dehydrogenase	*P. acidilactici, P. pentosaceus, P. parvulus, P. argentinicus, P. ethanolidurans, Pediococcus* sp.	Zhang et al., [117]
	*nox*	NADH oxidase	*P. acidilactici, P. pentosaceus, Pediococcus* sp.*, P. argentinicus, P. claussenii, P. cellicola, P. damnosus, P. ethanolidurans, P. inopinatus, P. parvulus, P. siamensis, P. stilesii*	Bryukhanov et al., [19]
	*npr*	NADH peroxidase	*P. acidilactici, P. claussenii, P. damnosus, P. parvulus, P. pentosaceus, Pediococcus* sp., *P. argentinicus, P. ethanolidurans, P. stilesii*	Bryukhanov et al., [19]
	*ohrR*	Organic hydroperoxide resistance transcriptional regulator	*P. cellicola, P. parvulus, P. siamensis, Pediococcus* sp.	Naraki et al., [66]
	*pox5*	Pyruvate oxidase	*P. acidilactici, P. pentosaceus, P. cellicola, P. damnosus, P. ethanolidurans, P. inopinatus, P. parvulus, P. siamensis, Pediococcus* sp.*, P. argentinicus, P. claussenii, P. stilesii*	[57]
	*thiO*	Thiamine biosynthesis oxidoreductase ThiO	*P. damnosus, P. inopinatus, Pediococcus* sp.*, P. claussenii, P. siamensis, P. cellicola, P. ethanolidurans, P. parvulus,*	Stetina et al., [94]
	*tpx*	Thiol peroxidase	*P. acidilactici, P. argentinicus, P. cellicola, P. claussenii, P. damnosus, P. ethanolidurans, P. inopinatus, P. parvulus, P. pentosaceus, P. siamensis, Pediococcus* sp.*, P. stilesii*	Bryukhanov et al., [19]
	*trxA*	Thioredoxin	*P. acidilactici, P. argentinicus, P. cellicola, P. claussenii, P. damnosus, P. ethanolidurans, P. inopinatus, P. parvulus, P. pentosaceus, P. siamensis, Pediococcus* sp.*, P. stilesii*	Bryukhanov et al., [19]
	*trxB*	Thioredoxin reductase	*P. acidilactici, P. argentinicus, P. cellicola, P. claussenii, P. damnosus, P. ethanolidurans, P. inopinatus, P. parvulus, P. pentosaceus, Pediococcus* sp.*, P. stilesii, P. siamensis*	Bryukhanov et al., [19]
	*ydaP*	Pyruvate oxidase	*P. acidilactici, P. ethanolidurans, P. pentosaceus*	Yu, Ye [114]
	*ytpP*	Thioredoxin-like protein YtpP	*P. acidilactici, P. argentinicus, P. cellicola, P. damnosus, P. ethanolidurans, P. inopinatus, P. parvulus, P. siamensis, Pediococcus* sp.*, P. claussenii, P. stilesii*	Pham et al., [75]
Other stress tolerance	*clpB*	Chaperone protein ClpB	*P. acidilactici, P. damnosus, P. ethanolidurans, P. inopinatus, P. parvulus, P. pentosaceus, P. siamensis, Pediococcus* sp.*, P. stilesii, P. cellicola*	Yang et al., [112]
	*clpE*	ATP-dependent Clp protease ATP-binding subunit ClpE	*P. acidilactici, P. argentinicus, P. cellicola, P. claussenii, P. damnosus, P. ethanolidurans, P. inopinatus, P. parvulus, P. siamensis, Pediococcus* sp.*, P. pentosaceus, P. stilesii*	Queraltó et al., [81]
	*clpL*	ATP-dependent Clp protease ATP-binding subunit ClpL	*P. acidilactici, P. pentosaceus, Pediococcus* sp.	Queraltó et al., [81]
	*clpP*	ATP-dependent Clp protease proteolytic subunit	*P. acidilactici, P. argentinicus, P. cellicola, P. claussenii, P. damnosus, P. ethanolidurans, P. inopinatus, P. parvulus, P. pentosaceus, P. siamensis, Pediococcus* sp.*, P. stilesii*	Queraltó et al., [81]
	*clpX*	ATP-dependent Clp protease ATP-binding subunit ClpX	*P. acidilactici, P. argentinicus, P. cellicola, P. claussenii, P. damnosus, P. ethanolidurans, P. inopinatus, P. parvulus, P. pentosaceus, P. siamensis, Pediococcus* sp.*, P. stilesii*	Queraltó et al., [81]
	*ctsR*	Transcriptional regulator CtsR	*P. acidilactici, P. argentinicus, P. cellicola, P. claussenii, P. damnosus, P. ethanolidurans, P. inopinatus, P. parvulus, P. pentosaceus, P. siamensis, Pediococcus* sp.*, P. stilesii*	Zhao et al., [121]
	*dnaK*	Chaperone protein DnaK	*P. acidilactici, P. argentinicus, P. cellicola, P. claussenii, P. damnosus, P. ethanolidurans, P. inopinatus, P. parvulus, P. pentosaceus, Pediococcus* sp.*, P. stilesii, P. siamensis*	(Bucka-Kolendo, et al. 2021)
	*ftsH*	ATP-dependent zinc metalloprotease FtsH	*P. acidilactici, P. argentinicus, P. cellicola, P. claussenii, P. damnosus, P. ethanolidurans, P. inopinatus, P. parvulus, P. pentosaceus, Pediococcus* sp.*, P. stilesii, P. siamensis*	Biswas et al., [14]
	*grpE*	Protein GrpE	*P. acidilactici, P. argentinicus, P. pentosaceus, P. stilesii, P. cellicola, P. damnosus, P. ethanolidurans, P. claussenii, P. inopinatus, P. parvulus, P. siamensis, Pediococcus* sp.	Lee et al., [49]
	*hrcA*	Heat-inducible transcription repressor HrcA	*P. acidilactici, P. sp., P. stilesii, P. cellicola, P. damnosus, P. ethanolidurans, P. inopinatus, P. parvulus, P. siamensis, Pediococcus* sp.*, P. argentinicus, P. claussenii*	Li et al., [52]
Vitamin biosynthesis	*folA*	Dihydrofolate reductase	*P. acidilactici, P. pentosaceus, P. argentinicus*	Mahara et al., [63]
	*folC*	Tetrahydrofolate synthase	*P. acidilactici, P. pentosaceus, Pediococcus* sp.*, P. claussenii, P. damnosus, P. cellicola, P. ethanolidurans, P. parvulus, P. stilesii, P. argentinicus*	Mahara et al., [63]
	*folE*	GTP cyclohydrolase 1	*P. acidilactici, P. cellicola, P. damnosus, P. ethanolidurans, P. inopinatus, P. parvulus, P. siamensis, Pediococcus* sp.*, P. stilesii, P. claussenii*	Mahara et al., [63]
	*folK*	2-amino−4-hydroxy−6-hydroxymethyldihydropteridine pyrophosphokinase	*P. acidilactici, P. pentosaceus, P. claussenii, P. stilesii, P. parvulus, P. damnosus, P. ethanolidurans, P. cellicola*	Mahara et al., [63]
	*folP*	Dihydropteroate synthase	*P. acidilactici, P. parvulus, P. stilesii, P. claussenii, Pediococcus* sp.*, P. pentosaceus*	Mahara et al., [63]
	*menA*	1,4-dihydroxy−2-naphthoate octaprenyltransferase	*P. pentosaceus*	Yang et al., [113]
	*menB*	1,4-dihydroxy−2-naphthoyl-CoA synthase	*P. pentosaceus, P. cellicola, P. parvulus, Pediococcus* sp.	Yang et al., [113]
	*menC*	o-succinylbenzoate synthase	*P. acidilactici, Pediococcus* sp.*, P. pentosaceus, P. cellicola, P. ethanolidurans, P. siamensis*	Yang et al., [113]
	*menD*	2-succinyl−5-enolpyruvyl−6-hydroxy−3-cyclohexene−1-carboxylate synthase	*P. pentosaceus*	Yang et al., [113]
	*menE*	2-succinylbenzoate--CoA ligase	*P. pentosaceus*	Yang et al., [113]
	*menF*	Isochorismate synthase MenF	*P. pentosaceus*	Yang et al., [113]
	*menH*	2-succinyl−6-hydroxy−2,4-cyclohexadiene−1-carboxylate synthase	*P. acidilactici, P. cellicola, P. claussenii, P. damnosus, P. ethanolidurans, P. inopinatus, P. parvulus, P. pentosaceus, P. siamensis, Pediococcus* sp.	Yang et al., [113]
	*panB*	3-methyl−2-oxobutanoate hydroxymethyltransferase	*P. acidilactici*	Zhao et al., [122]
	*ribB*	3,4-dihydroxy−2-butanone 4-phosphate synthase	*P. acidilactici, P. pentosaceus, P. stilesii, P. argentinicus, P. damnosus, P. parvulus, P. cellicola*	Wu, Zhang [108]
	*ribD*	Riboflavin biosynthesis protein RibD	*P. acidilactici, P. pentosaceus, P. stilesii, P. damnosus, P. parvulus, P. cellicola, P. ethanolidurans, P. siamensis*	Wu, Zhang [108]
	*ribE*	Riboflavin synthase	*P. acidilactici, P. stilesii, P. argentinicus, P. siamensis, P. pentosaceus, Pediococcus* sp.	Wu, Zhang [108]
	*ribF*	Bifunctional riboflavin kinase/FMN adenylyltransferase	*P. acidilactici, P. pentosaceus, Pediococcus* sp.*, P. cellicola, P. damnosus, P. ethanolidurans, P. inopinatus, P. parvulus, P. siamensis, P. claussenii, P. argentinicus, P. stilesii*	Wu, Zhang [108]
	*ribH*	6,7-dimethyl−8-ribityllumazine synthase	*P. acidilactici, P. stilesii, P. pentosaceus, Pediococcus* sp.*, P. argentinicus, P. siamensis*	Wu, Zhang [108]
	*thiD*	Hydroxymethylpyrimidine/phosphomethylpyrimidine kinase	*P. argentinicus, P. claussenii*	Xia et al., [110]
	*thiE*	Thiamine-phosphate synthase	*P. acidilactici, P. pentosaceus, Pediococcus* sp.*, P. stilesii, P. claussenii*	Xia et al., [110]
	*thiG*	Thiazole synthase	*P. acidilactici*	Xia et al., [110]
	*thiM*	Hydroxyethylthiazole kinase	*P. acidilactici, P. pentosaceus, Pediococcus* sp.*, P. stilesii, P. claussenii*	Xia et al., [110]

**Fig. 10:**
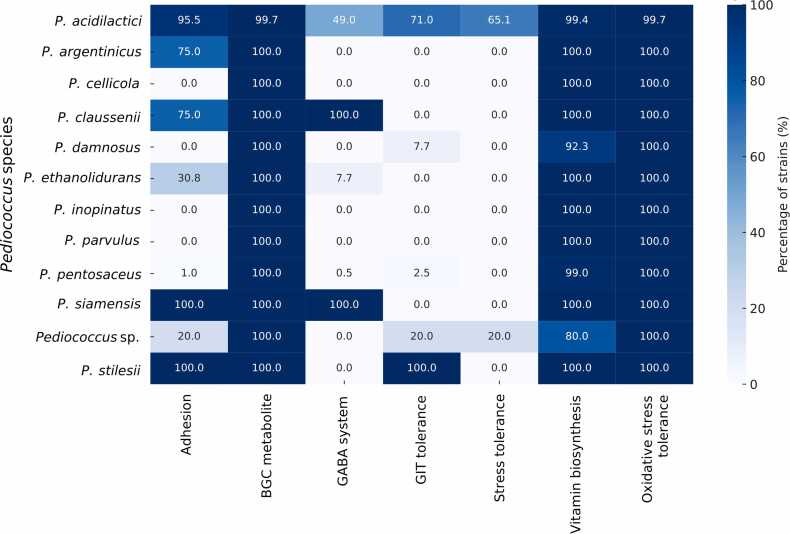
Distribution of probiotic-related and biosynthetic gene categories across *Pediococcus* species.

Comparative statistical analysis revealed significant differences in the prevalence of probiotic gene categories across *Pediococcus* species (Fisher’s exact test, q < 0.05). *P. acidilactici* exhibited broad functional enrichment, with significant prevalence of adhesion (95.5 %), GIT tolerance (71.0 %), oxidative stress tolerance (65.1 %), and the GABA system (49.0 %). These categories were significantly more abundant in *P. acidilactici* compared to other species (q < 0.05). In contrast, *P. pentosaceus* showed depletion in many categories, particularly adhesion and oxidative stress tolerance (OR = 0.00, q < 1e-93), but retained minimal enrichment in GIT tolerance (OR = 0.02, q < 1e-48). *P. parvulus* displayed weak enrichment across adhesion, GIT tolerance, and oxidative stress resistance, suggesting a lower functional capacity for these traits. Notably, *P. claussenii* was highly enriched in the GABA system (OR = ∞, q = 0.0258), indicating its specialization in this pathway. Vitamin biosynthesis genes were universally present in nearly all species, with no significant differences observed. In contrast, bacteriocin and secondary metabolite biosynthesis genes were present across all species without significant variation. Overall, *P. acidilactici* exhibited the most robust functional clustering, suggesting its broad probiotic potential, while other species like *P. pentosaceus* and *P. parvulus* exhibited more limited enrichment in key probiotic functions (Fig. 10 and Table S2).

## 4 Discussion

The genus *Pediococcus* holds considerable significance in food, health, and industrial microbiology. As a member of the LAB, *Pediococcus* contributes substantially to the fermentation of vegetables, meat, cereals, and beverages, where its acidification ability improves food safety, shelf life, and flavor [88]. Several species, particularly *P. acidilactici* and *P. pentosaceus*, are recognized for their probiotic potential, that have led to their application in functional foods, dietary supplements, and livestock feed [26], [79], [85]. In this study, the large-scale comparative genomic analysis of 616 *Pediococcus* genomes provides the most comprehensive overview to date of the taxonomy, genetic diversity, functional capacity, and probiotic potential of the genus. The findings integrate genomic composition, species delineation, accessory genome diversity, and multiple functional categories, including antimicrobial resistance, virulence, secondary metabolite biosynthesis, CAZymes, and probiotic-associated genes, thereby offering a holistic picture of the genus’s evolutionary and applied potential. Genome size analysis showed that *Pediococcus* species ranged from 0.56 to 2.53 Mbp, with most *P. acidilactici* and *P. pentosaceus* clustering between 1.8 and 2.1 Mbp, consistent with the compact genomes typical of LAB, adapted to nutrient-rich niches such as fermented foods and host environments [45], [72]. However, the low genome size values originated from metagenome-assembled genomes (MAGs), which often represent incomplete or fragmented assemblies, since they are derived from environmental samples rather than pure cultures. The distinct GC content profiles, *P. pentosaceus* around 37 % versus *P. acidilactici* between 41 % and 43 %, corroborate earlier reports of species-level divergence [115], [31], [53]. ANI analysis confirmed tight clades for *P. pentosaceus* and *P. acidilactici*, while *P. parvulus*, *P. ethanolidurans*, and *P. damnosus* formed separate groups with intra-species ANI > 95 %. Importantly, a subgroup of 13 *P. acidilactici* strains displayed approximately 90 % ANI and 37–38 % dDDH values relative to the type strains, below species-level thresholds (95 % ANI and 70 % dDDH). This genomic separation, supported by a distinct branch in the pan-genome phylogenomic tree, suggests a genetically unique sublineage, potentially representing a novel species within the *P. acidilactici* complex [86]. Pan-genome analysis revealed 34,011 orthologous clusters, with only 32 genes conserved across ≥ 99 % of genomes, confirming an open pan-genome and high genomic plasticity in the *Pediococcus* genus. This extremely small hard core reflects extensive accessory diversity and frequent gene turnover, consistent with LAB adaptation to diverse ecological niches, including plant materials, dairy, meat, alcoholic fermentations, and gut environments [120], [17]. The prevalence of strain-specific cloud genes explains why many ARGs, VFs, bacteriocins, and CAZymes are not universally distributed but occur sporadically across the dataset. This pattern also indicates that horizontal gene transfer (HGT) plays a key role in shaping the open nature of the *Pediococcus* pan-genome, contributing to genomic diversification and functional innovation. Such genetic exchanges likely facilitate the acquisition of adaptive traits, including carbohydrate utilization, bacteriocin synthesis, and environmental stress tolerance, enhancing the ecological versatility of the genus while maintaining overall genomic stability [18]. Only 41 of 616 strains (6.66 %) carried ARGs, confirming the low prevalence of resistance genes in *Pediococcus*. The most frequently detected ARGs were *tet(M)*, *ermB*, and *lnuA,* which encode well-characterized resistance mechanisms in LAB. These genes, along with *poxtA, fexB, vatE,* and *aph(3′)-IIIa*, are recognized as acquired ARGs typically located on mobile genetic elements (MGEs) such as plasmids or transposons, suggesting environmental acquisition under antibiotic selection pressure [104], [36], [67], [83], [93]. Their sporadic detection and restriction to a few strains, particularly those of animal origin (e.g., HC550 and HC128, each carrying nine ARGs), suggest environmental acquisition driven by antibiotic selection pressure rather than stable genomic inheritance within the genus. In contrast, intrinsic, chromosomally encoded genes such as *cat-TC* (chloramphenicol acetyltransferase) and low-identity efflux pumps were more broadly distributed and not associated with horizontal transfer [61]. According to the EFSA BIOHAZ Panel statement on the interpretation of acquired antimicrobial resistance genes in Qualified Presumption of Safety (QPS) microorganisms [35], such intrinsic determinants are not considered a safety concern. Therefore, the predominance of intrinsic and non-mobile resistance genes, combined with the low frequency and non-conserved presence of acquired ARGs, supports that *Pediococcus* poses minimal risk for antimicrobial resistance dissemination and remains genomically safe for probiotic and food applications. In addition, virulence profiling showed that 615 of 616 genomes (99.84 %) carried at least one VF-annotated gene. However, the most prevalent hits were *clpP, tufA, gndA,* and *hasC*, which are primarily housekeeping and stress-associated functions rather than classical virulence determinants for probiotic traits. The *clpP* encodes the proteolytic subunit of the Clp protease complex, which is essential for protein quality control, heat shock response, and cellular stress tolerance; although in some pathogenic bacteria it influences virulence regulator turnover, its primary role in LAB is proteostasis and environmental resilience [9]. TufA, which encodes elongation factor Tu, is vital for translation and also functions as a moonlighting adhesin in certain LAB, aiding mucin binding and gut colonization rather than pathogenesis [29]. GndA encodes 6-phosphogluconate dehydrogenase, a key enzyme in the pentose phosphate pathway that provides NADPH for redox balance and biosynthesis [37]. HasC encodes UDP-glucose pyrophosphorylase, providing activated sugars for cell wall and capsule polysaccharides, contributing to cell integrity and stress protection [59]. These genes are nearly universal in lactic acid bacteria and are essential for survival, not virulence. Their annotation as virulence factors reflects sequence homology to pathogenic counterparts, but not equivalent biological function. This highlights an important limitation of VFDB annotation, where functional overlap between metabolic or stress-related proteins and virulence-associated homologs can lead to overprediction of virulence genes in non-pathogenic taxa. Consistent with previous LAB studies, *Pediococcus* exhibits no true pathogenic determinants, and the VFDB matches identified here represent non-pathogenic homologs that support cellular maintenance, environmental adaptation, and potential probiotic functions [119], [39], [82]. The low prevalence of ARGs and absence of mobile virulence elements in this finding confirm the genomic safety and non-pathogenic nature of the *Pediococcus* genus. Functional annotation of secondary metabolite and bacteriocin biosynthetic clusters revealed key traits with direct application in food preservation. Secondary metabolite mining revealed widespread terpene, RiPP, NRPS, PKS, and T3PKS clusters. RiPPs were more abundant than NRPS, and *P. pentosaceus* carried more PKS and T3PKS clusters than *P. acidilactici*. RiPPs include bacteriocins, which were abundant in the dataset. The distribution of complete bacteriocin BGCs among *Pediococcus* species revealed a highly uneven and species-specific pattern. The presence of bacteriocin clusters was low across the genus, with most species carrying few or none. *P. pentosaceus* and *P. acidilactici* displayed the greatest diversity of bacteriocins, though still at low frequencies, including clusters encoding pediocin, AFP-1, bovicin 255, enterolysin A, and penocin A. In particular, pediocin clusters, belonging to the class IIa bacteriocins known for strong anti-*Listeria* activity, were detected in *P. pentosaceus* and *P. acidilactici* genomes [116], [44]. These bacteriocins are heat-stable, highly conserved peptides that play a central role in the biopreservative potential of *Pediococcus*, explaining their use in meat and dairy fermentations [80]. Certain species showed distinctive enrichment patterns. *P. damnosus* exhibited the highest proportion of complete pediocin clusters, indicating a potentially strong antimicrobial capability within this lineage. *P. stilesii* harbored carnocin CP52 and propionicin F clusters in half of its genomes, suggesting a narrower but more specialized bacteriocin profile. Meanwhile, *P. cellicola* and *P. claussenii* carried bovicin 255 and carnocin CP52 clusters, and *P. argentinicus* contained a putative bacteriocin cluster. By contrast, several species, such as *P. parvulus* and *P. inopinatus,* showed only sporadic occurrence of bacteriocin clusters, and no complete clusters were detected in *P. ethanolidurans*, *P. siamensis*, or unclassified *Pediococcus* spp. This uneven distribution suggests substantial genomic diversity in bacteriocin biosynthetic capacity among *Pediococcus* species, which may influence their ecological niches and antimicrobial potential. Another important characteristic of the LAB group is carbohydrate-active enzymes for plant- and food-matrix adaptation. CAZyme profiling revealed dominance of GH and GT, followed by CBM and carbohydrate esterases CE. The most prevalent families were GH1, GH13, GH43, GT2, GT4, and GT8. The most prevalent CAZyme families included GH1, GH13, GH43, GT2, GT4, and GT8, each contributing to critical metabolic functions related to carbohydrate degradation and biosynthesis. The GH1 (β-glucosidases) family contributes to the hydrolysis of β-linked glycosides, facilitating the release of glucose and flavor-active compounds during fermentation [65], while the GH13 (α-amylases) family targets starch and glycogen to generate maltose and glucose, thereby enhancing lactic acid production and improving fermentation kinetics in cereal- and vegetable-based fermentations. Similarly, the GH43 (xylosidases and arabinofuranosidases) family degrades arabinoxylans and hemicelluloses, enabling nutrient acquisition from complex plant polysaccharides [89]. In addition to these general fermentation roles, several GH families are associated with the metabolism of indigestible dietary fibers such as prebiotics, linking CAZyme potential to probiotic functionality [46]. Importantly, the identification of GH32 and GH91 families in several species supports the genomic capacity to utilize fructo-oligosaccharides (FOS), such as inulin. GH32 enzymes, including endo- and exo-inulinases, hydrolyze β-2,1-glycosidic linkages of inulin to release fermentable fructose and short-chain fructans, while GH91 inulin lyases cleave inulin polymers via β-elimination to yield oligosaccharides that feed into central carbon pathways [40], [90]. Other GH families, including GH23, GH24, GH25, GH38, and GH70, occurred sporadically and appear to be species-specific, suggesting functional diversification linked to distinct environmental pressures. Moreover, the GT2, GT4, and GT8 families catalyze the polymerization of sugar residues into cell wall components and exopolysaccharides (EPS), which are critical for biofilm formation, stress tolerance, and texture development in fermented foods [106], [3]. Such EPS production not only enhances fermentation performance and product viscosity but also contributes to protective barrier formation, as exemplified by *P. parvulus* strains producing β-glucan EPS in wine [27]. The presence of these CAZymes supports the adaptation of *Pediococcus* species to carbohydrate-rich niches such as cereals, vegetables, and alcoholic fermentations. Furthermore, a clear relationship was observed between CAZyme richness and the ecological origin of isolates, providing deeper insight into their environmental adaptation. Strains isolated from traditional fermented food sources, such as *P. acidilactici, P. pentosaceus*, and *P. damnosus*, exhibited broader CAZyme repertoires and a higher abundance of GH and GT families compared to those derived from fecal or beverage-associated environments, such as *P. stilesii* and *P. claussenii*, which showed narrower enzymatic profiles and reduced representation of CE, AA, and PL families. These patterns suggest that carbohydrate availability in the environment exerts selective pressure on CAZyme diversity, with food-associated strains retaining a wider range of enzymes to degrade complex polysaccharides and metabolize diverse sugar sources.

Moreover, the identification of probiotic-associated genes is widely distributed across the genus, but with notable species-specific functional patterns. Adhesion capacity was strongly enriched in *P. acidilactici* and *P. claussenii*, supported by the presence of *cna* (collagen adhesin) and *srtA* (sortase A). These genes are central to the anchoring of LPXTG-motif proteins on the cell wall, enhancing mucosal adhesion and colonization potential. Such mechanisms have been reported as critical for epithelial interaction in LAB, reinforcing the probiotic potential of these species [103], [70]. GABA production is the crucial criterion for selecting probiotic strains with potential functional food applications, which provide neuroactive benefits that contribute to host health [84]. The GABA system is encoded by the glutamate decarboxylase (GAD) operon and comprises two key components: *gadA* and *gadB*, which encode glutamate decarboxylase isozymes that catalyze the conversion of glutamate into GABA, while exporting the product in exchange for substrate import. This decarboxylation reaction consumes cytoplasmic protons, contributing to acid resistance and intracellular pH homeostasis, while generating GABA as a neuroactive metabolite with potential health benefits [41]. In addition, *puuD*, which encodes γ-glutamyl-GABA hydrolase, participates in downstream GABA metabolism, enabling further utilization of GABA within the cell [48]. In our analysis, the complete GAD system was frequently detected in *P. acidilactici*, whereas only *gadC* was found in several other *Pediococcus* species, suggesting partial retention or alternative pathways for GABA metabolism as reported in other LABs [23], [47]. GABA-producing capabilities have been widely described in other LABs, such as *Lactiplantibacillus plantarum* [43]. Genes associated with GIT tolerance were well represented, particularly the arginine deiminase (ADI) pathway (*arcA, arcB, arcD*) contributes to acid resistance through ammonia production, while the F_1_F_0_-ATPase complex (*atpA-H*) actively extrudes protons to maintain intracellular pH homeostasis under acidic environments [38]. The D-alanylation of teichoic acids (*dltA-D*) modifies cell wall charge, enhancing resistance to bile salts and antimicrobial peptides. The *bshA* was broadly distributed, while *bshB1* was unique to *P. damnosus*, reflecting lineage-specific bile salt hydrolase diversity. The higher prevalence of acid/bile tolerance determinants in *P. acidilactici* compared with *P. damnosus* aligns with its broader ecological distribution and established probiotic applications [50], [91], [96]. Oxidative stress response genes (e.g., *nox, npr, katA, tpx, msrA/msrB, trxA/trxB*) were enriched in *P. acidilactici*, providing defense against reactive oxygen species generated during food processing or gut transit [5], [50]. Furthermore, core stress response regulators (*clpP, dnaK, hrcA, ftsH*) were detected in higher than 92 % of strains, reflecting highly conserved proteostasis systems essential for survival under environmental fluctuations [20,95]. Vitamin biosynthesis pathways (riboflavin: *ribA-H*; folate: *folA-K*; thiamine: *thiD-M*) were nearly universal (>99 %), suggesting that *Pediococcus* species possess a strong biosynthetic capacity for nutritional fortification in fermented foods. These metabolic capabilities align with reports of LAB strains used in food biofortification for enhanced riboflavin and folate production [34], [99]. Significant functional specialization among *Pediococcus* species exhibited broad probiotic potential, while others showed more specialized traits. *P. acidilactici* exhibited the strongest functional clustering, with high prevalence of adhesion (95.5 %), GIT tolerance (71.0 %), oxidative stress tolerance (65.1 %), and the GABA system (49.0 %), indicating its broad probiotic potential. In contrast, *P. pentosaceus* showed depletion in adhesion and oxidative stress tolerance, with only minimal enrichment in GIT tolerance, suggesting a more specialized role. *P. claussenii* was highly enriched in the GABA system, positioning it as a candidate for neuroactive applications [25]. Other species, such as *P. parvulus, P. damnosus,* and *P. ethanolidurans*, showed limited functional clustering, implying a narrower probiotic profile. Bacteriocin and vitamin biosynthesis genes were universally present across species, underscoring their role in microbial competition and fermentation [4]. The functional clustering scores provide a deeper insight into the functional richness of *Pediococcus* species. For example, the high functional clustering score (318.32) of *P. acidilactici* reflects its broad functional capacity, which may translate into greater versatility in probiotic applications, including those targeting gut health, immune modulation, and stress resilience. In contrast, the low functional clustering score (1.97) for *P. inopinatus* suggests that it may have more narrowly defined probiotic properties, potentially useful in specific niches rather than as a broad-spectrum probiotic. The analysis revealed that *P. acidilactici* exhibited the broadest-spectrum probiotic traits, making it a promising candidate for gut health and stress resilience. Future research could further investigate the synergistic interactions of these functional traits in gastrointestinal and systemic health contexts. While this study provides comprehensive genomic insights into the distribution and functional potential of *Pediococcus* species, it is important to note that these findings are derived solely from silico analyses. Experimental validation, such as transcriptomic, proteomic, or phenotypic assays, will be essential to confirm whether the identified genes are actively expressed and contribute to the observed probiotic or metabolic traits. Nonetheless, the genomic framework established here offers valuable guidance for future investigations aiming to characterize and harness the functional capabilities of *Pediococcus* in food, health, and biotechnological applications.

## 5 Conclusion

The comparative genomic analysis of 616 *Pediococcus* strains reveals a genus characterized by low antimicrobial resistance, minimal virulence potential, and broad functional diversity. Core traits such as bacteriocin production, carbohydrate-active enzymes, stress tolerance, and vitamin biosynthesis were widely distributed, while species-specific features, such as strong probiotic potential in *P. acidilactici*, rich bacteriocin repertoires in *P. pentosaceus*, and niche-specific adaptations in minor species, highlight the ecological versatility and applied value of this genus. These findings provide a genomic framework supporting the safety and utility of *Pediococcus* as starter cultures, probiotics, and reservoirs of biofunctional traits for food and biotechnological innovation.

## CRediT authorship contribution statement

**Nattarika Chaichana:** Data curation, Formal analysis, Investigation, Methodology, Software, Validation, Writing – original draft. **Komwit Surachat:** Conceptualization, Funding acquisition, Methodology, Project administration, Resources, Supervision, Validation, Writing – review & editing. **Kamonnut Singkhamanan:** Funding acquisition, Methodology, Resources. **Sirikan Suwannasin:** Formal analysis, Investigation, Software. **Thitaporn Dechathai:** Formal analysis, Investigation, Software. **Jirasa Boonsan:** Investigation, Resources. **Rattanaruji Pomwised:** Methodology, Resources, Supervision. **Monwadee Wonglapsuwan:** Conceptualization, Methodology, Resources.

## Declaration of Competing Interest

The authors declare no conflicts of interest.

## Data Availability

Data will be made available on request.
