# Supplementary material for: Comparative genomic analysis of the Pediococcus genus reveals functional diversity for fermentation and probiotic applications
Source: Comput Struct Biotechnol J. 2025 Oct 25;27:4597–614. doi: 10.1016/j.csbj.2025.10.050 (PMC12613055; doi:10.1016/j.csbj.2025.10.050)
Supplement: Supplementary file 1 — Supplementary material [file mmc1.docx]

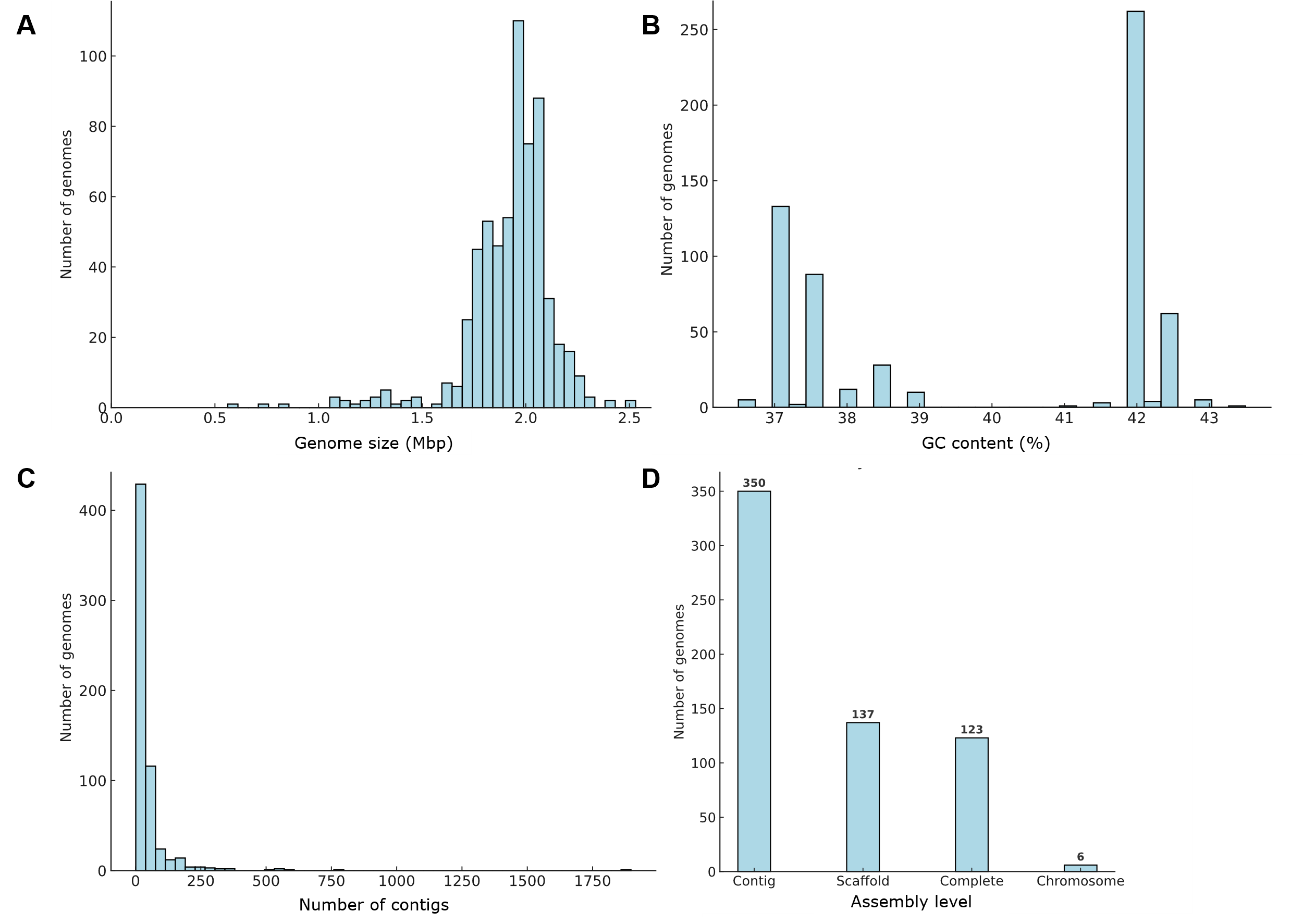


Figure S1. Genome information distribution among 616 *Pediococcus* strains available in the NCBI database. (A) Genome size distribution. (B) GC content distribution, ranging from 36.5% to 43.5%. (C) Number of contigs per genome, ranging from 1 to 1,897. (D) Assembly-level distribution, showing that most genomes were represented at the contig level.


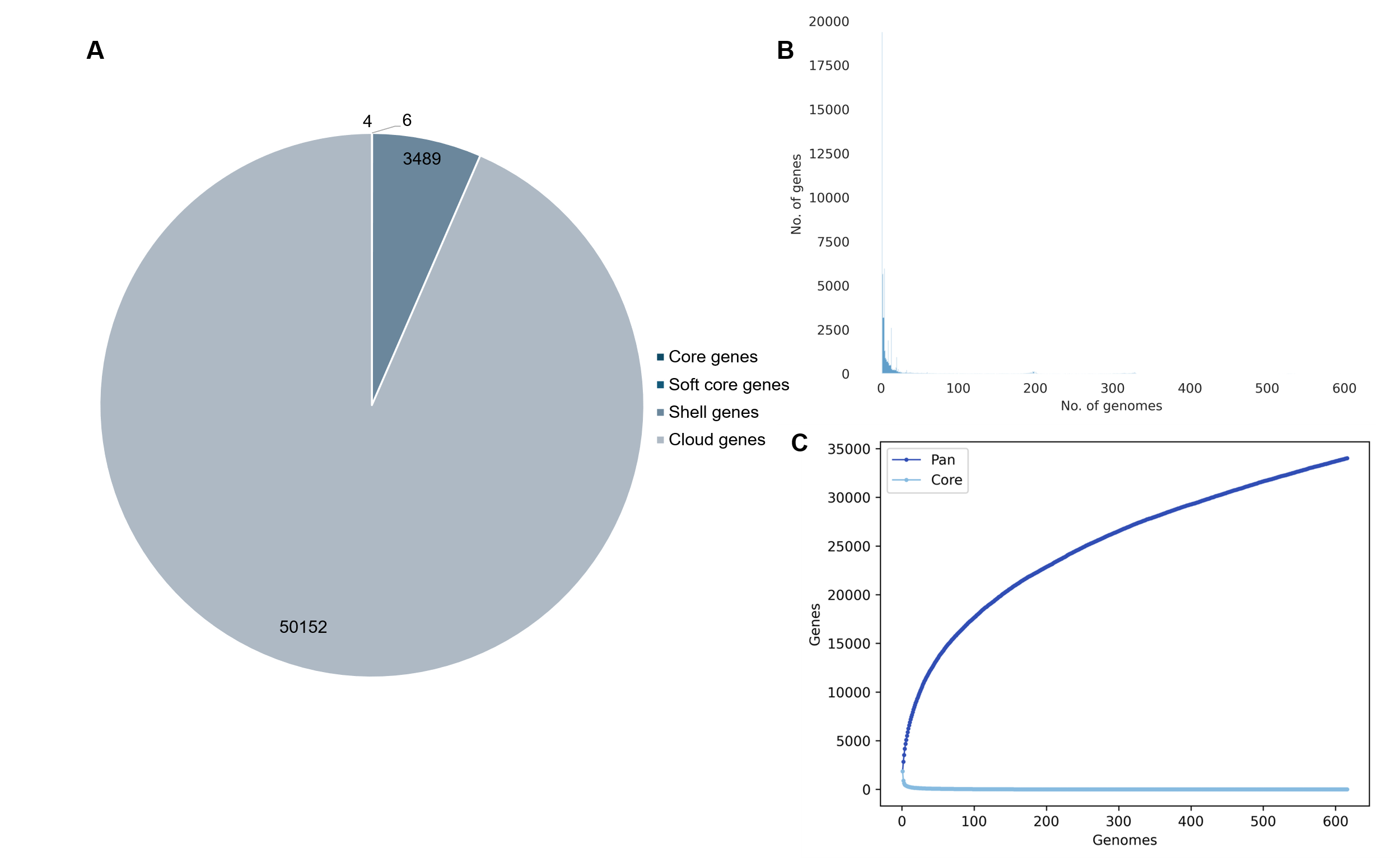


Figure S2. Pangenome overview of 601 *Pediococcus* genomes. (A) Pie chart showing that most genes are cloud (50,152) and shell (3,489), with only 4 core and 6 soft-core genes detected. (B) Gene frequency distribution highlighting the dominance of rare, strain-specific genes. (C) Pan-genome accumulation curve showing continuous gene gain and a near-zero core genome, indicating an open pangenome.Table S1 Digital DNA-DNA hybridization (dDDH) values between the potential novel *Pediococcus* lineage and reference *P. acidilactici* strains.

| Accession ID | Strain | Closest reference strain | dDDH value (%) |
| --- | --- | --- | --- |
| GCA_009809575.1 | FAM18987 | *P. acidilactici* JCM 8797 | 37.0 |
| GCA_024970065.1 | SRCM210477 | *P. acidilactici* JCM 8797 | 37.2 |
| GCA_036431145.1 | FI11617 | *P. acidilactici* DSM 20284 | 37.8 |
| GCA_036431165.1 | FI11626 | *P. acidilactici* DSM 20284 | 38.4 |
| GCA_036431185.1 | FI11639 | *P. acidilactici* JCM 8797 | 38.4 |
| GCA_036431205.1 | FI11636 | *P. acidilactici* DSM 20284 | 37.6 |
| GCA_036431225.1 | FI11591 | *P. acidilactici* JCM 8797 | 38.1 |
| GCA_036431285.1 | FI11602 | *P. acidilactici* DSM 20284 | 38.4 |
| GCA_036431875.1 | LMAN-Df | *P. acidilactici* DSM 20284 | 38.2 |
| GCA_036431925.1 | CDOD-Kf | *P. acidilactici* DSM 20284 | 38.2 |
| GCA_036432065.1 | LAMZ-Ka | *P. acidilactici* DSM 20284 | 38.2 |
| GCA_036432075.1 | LAMZ-Dg | *P. acidilactici* JCM 8797 | 38.2 |
| GCA_036432185.1 | CDOD-Kg | *P. acidilactici* JCM 8797 | 38.2 |

Table S2. Functional enrichment category of probiotic-associated genes in *Pediococcus* species.

| Species | Significant categories (q<0.05) | Functional clustering score (Σ -log10 q) | Max OR (category) | Enriched set |
| --- | --- | --- | --- | --- |
| *P. acidilactici* | 4 | 318.32 | Adhesion | Adhesion (OR=354.96, q=4.45e-130),  GABA system (OR=37.76, q=2.42e-43),  GIT tolerance (OR=64.33, q=8.95e-73),  Oxidative stress tolerance (OR=262.16, q=5e-75) |
| *P. pentosaceus* | 4 | 222.52 | GIT tolerance | Adhesion (OR=0.00, q=4.9e-93),  GABA system (OR=0.01, q=4.62e-34),  GIT tolerance (OR=0.02, q=2.69e-48),  Oxidative stress tolerance (OR=0.00, q=5.01e-50) |
| *P. parvulus* | 4 | 12.43 | Adhesion | Adhesion (OR=0.00, q=4.25e-06),  GABA system (OR=0.00, q=0.0258),  GIT tolerance (OR=0.00, q=0.00083),  Oxidative stress tolerance (OR=0.00, q=0.00406) |
| *P. damnosus* | 2 | 5.22 | Adhesion | Adhesion (OR=0.00, q=0.000236),  Oxidative stress tolerance (OR=0.00, q=0.0258) |
| *P. ethanolidurans* | 2 | 3.43 | GIT tolerance | GIT tolerance (OR=0.00, q=0.0143),  Oxidative stress tolerance (OR=0.00, q=0.0258) |
| *P. inopinatus* | 1 | 1.97 | Adhesion | Adhesion (OR=0.00, q=0.0107) |
| *Pediococcus sp.* | 1 | 1.71 | Stress tolerance | Stress tolerance (OR=0.03, q=0.0193), |
| *P. claussenii* | 1 | 1.59 | GABA system | GABA system (OR=inf, q=0.0258) |
